# Anticariogenic Activity of Black Tea: An In Vivo Study

**DOI:** 10.7759/cureus.38460

**Published:** 2023-05-02

**Authors:** Pinky Goswami, Chandana Kalita, A.C. Bhuyan

**Affiliations:** 1 Department of Dentistry, Lakhimpur Medical College & Hospital, Lakhimpur, IND; 2 Department of Conservative Dentistry and Endodontics, Regional Dental College, Guwahati, IND

**Keywords:** epigallocatechin gallate, green tea, theaflavin, black tea, anticariogenic

## Abstract

Background: The prevention of dental caries has always remained a challenge. Caries prevention through dietary intervention holds promise. Studies have revealed that several constituents present in tea have anticariogenic properties. Tea is a widely consumed beverage and hence could be utilized as a suitable caries preventive agent. The purpose of this study was to determine the effect of black tea on caries progression in experimental animals.

Materials and methods: This study was carried out in 17-day-old albino rat pups. The animals were divided into three groups, with eight animals in each group. They were fed on a cariogenic diet (MIT 200) and inoculated with *Streptococcus mutans*. Group I was given MIT 200 with water, Group II was placed on MIT 200 with black tea, and Group III was placed on MIT 200 with fluoridated water for a period of 45 days. After 45 days, the animals were killed under ether anesthesia, and their teeth were examined for caries.

Results: The carious lesions were scored for the first two molars in each quadrant. In each group, a total of 64 teeth were examined. The caries score between the upper and lower jaws was compared using ANOVA.

Conclusion: From this study, it may be inferred that drinking black tea reduced the development of dental caries in young rats fed on a cariogenic diet. The tea used for this study was prepared using fluoride-free water, so we can assume that besides fluoride, certain components are present in tea leaves that possess anticariogenic properties.

## Introduction

Dental caries is a microbial disease associated with biofilm formation [1]. The oral biofilm is a complex structure of microorganisms present within an intercellular matrix derived from saliva, gingival crevicular fluid, and bacterial products. A delicate balance exists between the host tissues and the microbial population of the dental biofilm. However, this biofilm can become pathogenic because of an increase in the number of acidogenic bacteria, such as *Streptococcus mutans* (*S. mutans*), leading to a shift in the balance and causing an increase in demineralization, which ultimately leads to dental caries. The bacteria responsible for dental caries are mainly *S. mutans* for its initiation and *Lactobacillus* for its progression. The ability of *S. mutans* to generate biofilm on both soft and hard tissues, including the palate, tongue, and teeth, is correlated with its pathogenicity [1]. Glucosyltransferase is an enzyme produced by *S. mutans* to catalyze the synthesis of glucans from the metabolism of sucrose [2,3]. The glucans aid in the colonization of dental plaque by various bacteria, and glucosyltransferase plays an important role in the virulence of *S. mutans* by forming biofilm extracellular polysaccharides (EPS) [4]. This EPS matrix not only enhances mechanical stability but also enables bacterial adhesion to oral surfaces [5].

Tea is one of the most widely consumed beverages in the world. Tea is basically classified as green tea, oolong tea, or black tea based on its manufacturing process. During the fermentation process used in the production of black tea, catechins present in the tea leaf are converted into flavonoids [6,7]. Because green tea is non-fermented, it contains a high concentration of catechins. The principal catechins present in green tea are epigallocatechin 3-gallate (EGCG), epigallocatechin (EGC), epicatechin (EC), EC 3-gallate (ECG), and catechin (C) [7]. Numerous gram-positive bacteria, such as *Staphylococcus* species and *Streptococcus* species, and gram-negative bacteria, such as *Porphyromonas gingivalis* and *Treponema denticola,* have been reported to be inhibited by these polyphenols [8]. By inhibiting certain genes involved in the activation of glucosyltransferase, it has been demonstrated to interfere with the production of EPS and biofilm by *S. mutans* [9]. Quorum sensing, a process of intercellular communication used by several bacteria, can be disrupted by green tea polyphenols, particularly EGCG [10]. Studies have also shown that the consumption of black tea affects the initiation and progression of dental caries in experimental animals [11]. This study determined the anticariogenic properties of black tea.

## Materials and methods

**Figure 1:**
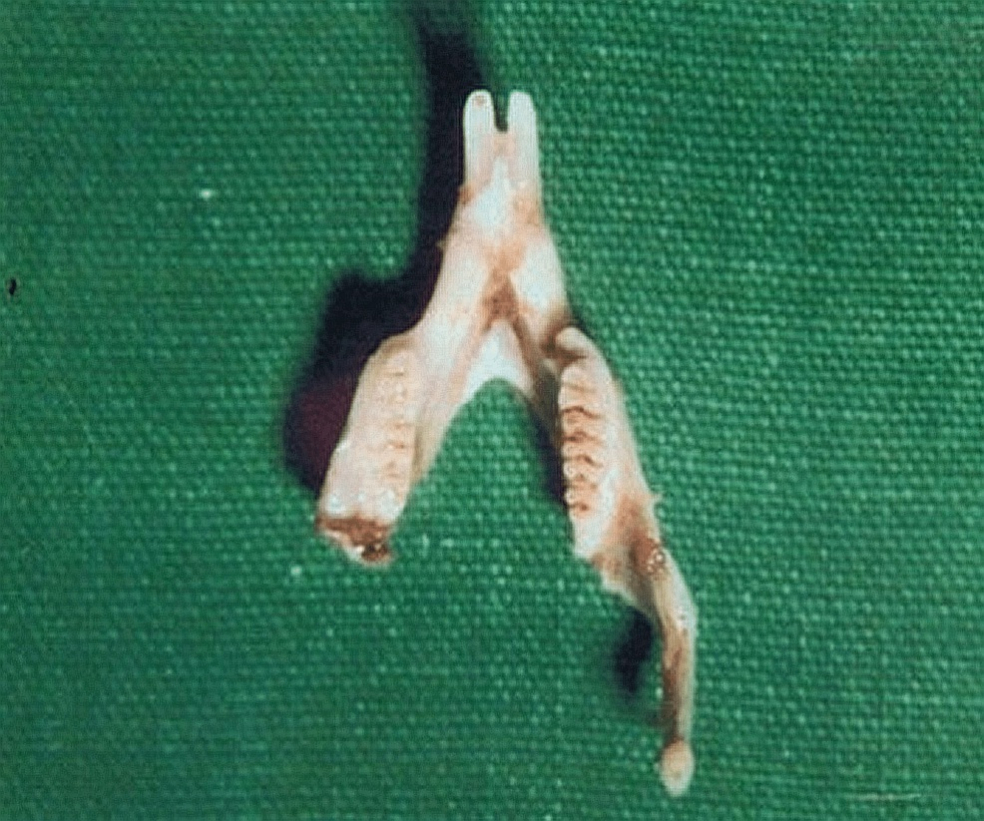
Mandible resected from rats

This study was carried out to determine whether the consumption of black tea influences the process of caries progression in young caries-prone experimental animals. This in vivo experimental study was carried out after obtaining due permission from the Institutional Ethics Committee (reference number: RDC/29/2011/2718). This experiment was carried out on albino rats, and four adult female rats were housed in one cage along with one male rat (Figure 1).

The female-to-male ratio was maintained at 4:1. The cages were lined with paddy husks and the animals were put on a diet of germinated grams, apples, and cabbages. After breeding, the pregnant female rats were placed separately in plastic cages lined with cellulose bedding (shredded paper). After a gestation period of about 21 days, the female rats littered. The newborn pups were left undisturbed with the mother for a period of 17 days. After 17 days, the pups were weaned and placed in separate cages. The animals were then segregated into three batches of eight each. The animals were placed on MIT 200 (cariogenic diet) [12]. An inoculum of *S. mutans *was mixed with the drinking water and supplied to all three groups of animals for five consecutive days. In Group I, the animals were fed on MIT 200 and water; in Group II, the animals were fed on MIT 200 and black tea infusion where black tea infusion was prepared following the recommendations of the manufacturers. Each tea bag sample was added separately to 90 ml of freshly boiled water and stirred. The tea bags were taken out after three minutes. In Group III, the animals were fed on MIT 200 and fluoridated water. The rats were placed on this test diet for a period of 45 days [11]. After 45 days, the animals were killed using ether anesthesia. The maxilla and mandible were dissected out and immersed in a solution of 10% formalin for five days. The remaining tissue debris was cleaned and the teeth were examined under a stereomicroscope for the presence of caries on the buccal, lingual, and proximal surfaces. The present study has been carried out on the more commonly available albino rats and we have used non-branded locally available Assam tea.

For a total of 64 teeth in each group, the first two molars in each quadrant were assessed for caries. The maxillary and mandibular molars were hemisected in a mesiodistal sagittal plane. The carious lesions limited to the enamel were scored as E, and those penetrating up to the dentin were scored as D. The caries were scored as 0, 1, 2, 3, and 4, where 0 denotes the absence of caries, 1 denotes caries of the enamel, 2 denotes a lesion with slight involvement of the dentin, 3 denotes lesions with moderate involvement, and 4 denotes extensive involvement of the dentin. The carious lesions, which progressed up to one-fourth of the dentin, i.e., the distance from the enamel to the pulp chamber, were assigned as slight; the lesions extending between one-fourth and three-fourths of this distance were classified as moderate; and the lesions extending beyond three-fourths of this distance were classified as severe. The caries score for each tooth is a sum total of the caries score obtained from the buccal, lingual, and sulcal lesions [13].

## Results

The caries scores in the maxilla and mandible were compared using ANOVA (Tables 1-3).

**Table 1:** Caries score for first and second molar teeth (Group I) Caries scored for first and second molar teeth are from buccal, lingual, and sulcal lesions.

Maxillary teeth		Mandibular teeth		P-value
1^st^ molar	2^nd ^molar	1^st^ Molar	2^nd ^Molar	
3.1	2.3	3.0	2.1	0.01
3.0	2.2	2.1	2.0	
3.1	3.2	3.0	3.0	
3.2	3.1	3.0	3.0	
3.2	3.3	3.0	3.2	
3.4	3.2	3.1	3.1	
3.1	3.2	3.1	3.0	
3.3	3.0	3.2	3.1	
3.2	3.0	3.1	3.0	
3.1	3.2	3.2	3.0	
3.1	3.0	3.1	3.0	
3.2	3.1	3.2	3.0	
3.0	3.0	3.2	3.2	
3.3	3.1	2.2	3.3	
3.2	3.4	3.0	2.4	
2.5	3.0	2.3	3.0	

**Table 2:** Caries score for first and second molar teeth (Group II) Caries scored for first and second molar teeth are from buccal, lingual, and sulcal lesions.

Maxillary teeth		Mandibular teeth		P-value
1^st^ molar	2^nd^ molar	1^st^ molar	2^nd^ molar	
1.4	0	1.2	1.1	0.01
1.4	1.3	1.4	1.2	
1.4	1.4	1.1	1.2	
0	1.4	1.3	1.2	
0	1.3	1.1	1.2	
1.4	1.2	1.3	1.1	
1.4	1.4	1.2	1.2	
1.3	1.4	0	1.0	
1.3	1.4	1.2	1.3	
1.4	1.3	1.2	1.0	
1.2	1.4	1.1	1.1	
1.2	1.4	1.2	1.1	
1.4	1.3	1.2	1.2	
1.2	1.4	1.1	1.0	
1.2	1.0	1.1	1.0	
1.3	1.2	1.1	1.2	

**Table 3:** Caries score for first and second molar teeth (Group III) Caries scored for first and second molar teeth are from buccal, lingual, and sulcal lesions.

Maxillary teeth		Mandibular teeth		P-value
1^st^ molar	2^nd^ molar	1^st^ molar	2^nd^ molar	
1.1	1.2	1.0	1.0	0.01
1.0	0	1.2	0	
1.0	1.0	1.2	1.0	
1.0	0	1.2	0	
0	0	0	0	
1.2	1.0	1.1	1.0	
0	0	0	0	
0	1.1	0	1.2	
1.2	1.0	1.0	0	
1.0	0	1.0	1.0	
0	0	1.0	0	
0	1.1	0	0	
1.0	1.2	1.0	1.1	
0	1.1	1.0	0	
1.0	0	0	1.0	
0	1.2	1.0	0	

A p-value < 0.01 was considered significant. The difference in total caries score among the three groups was found to be statistically significant (p < 0.01). The mean difference in the caries score between the maxilla and mandible in all the groups was found to be statistically significant. It was observed that the caries score was highest in Group I (receiving a cariogenic diet and non-fluoridated water). The caries score was comparatively higher in Group II (receiving black tea and a cariogenic diet) than in Group III (receiving fluoridated water and a cariogenic diet).

ANOVA was performed to compare the total caries score among the three groups (Table 4).

**Table 4:** Results of statistical analysis of different groups for caries score of first and second molar teeth

Group	Jaw	Mean	Standard error of the mean
Group I	Upper jaw	3.07	0.04
	Lower jaw	2.91	0.65
	Total	2.99	0.41
Group II	Upper jaw	1.22	0.60
	Lower jaw	1.12	0.03
	Total	1.17	0.03
Group III	Upper jaw	0.60	0.09
	Lower jaw	0.59	0.09
	Total	0.60	0.06

In Group I, the mean caries score of the upper jaw was found to be 3.07 with a standard error of 0.04 and of the lower jaw was found to be 2.91 with a standard error of 0.65. The overall mean of Group I was found to be 2.99 with a standard error of 0.41. In Group II, the mean caries score of the upper jaw was found to be 1.22 with a standard error of 0.60 and of the lower jaw was found to be 1.12 with a standard error of 0.03. The overall mean of Group II was found to be 1.17 with a standard error of 0.03. In Group III, the mean caries score of the upper jaw was found to be 0.60 with a standard error of 0.09 and of the lower jaw was found to be 0.59 with a standard error of 0.09. The overall mean of Group III was found to be 0.60 with a standard error of 0.06.

In Group I, the mean caries score of the maxillary teeth was found to be 3.07 with a standard error of 0.04, and in Group II, it was found to be 1.22 with a standard error of 0.60. The differences in mean values between Groups II and III are tested for statistical significance using analysis of variance. The results are summarized in Table 5.

**Table 5:** Results of one-way analysis of variance performed to compare the total caries score between Group I & Group II (maxillary teeth) F (1, 62) = 7.47; p < 0.01.

Sources of variation	Degrees of freedom (df)	Sum of squares (ss)	Mean square (MSS = SS/df)	F ratio = group MSS/error MSS
Between groups	1	52.74	52.74	7.47
Error	62	437.53	7.05	-
Total	63	490.28	-	-

In Group I, the mean caries score of mandibular teeth was found to be 2.91 with a standard error of 0.65, and in Group II, the mean caries score was found to be 1.12 with a standard error of 0.03. The differences in mean values between Groups I and II are tested for statistical significance using ANOVA. The results are summarized in Table 6.

**Table 6:** Results of one-way analysis of variance performed to compare the total caries score between Group I & Group II (mandibular teeth) F (1, 62) = 47.32; p < 0.01.

Sources of variation	Degrees of freedom (df)	Sum of squares (SS)	Mean squares (MSS = SS/df)	F ratio = group MSS/error MSS
Between groups	1	79.62	79.62	47.32
Error	62	104.31	1.68	-
Total	63	183.93	-	-

The difference in mean caries of Groups I and II in the maxillary teeth was found to be statistically significant, indicating that Group I maxillary teeth have more caries than Group II.

ANOVA was performed to compare the total caries score among the three groups and upper and lower jaw (Table 7).

**Table 7:** Results of one-way analysis of variance performed to compare the total caries score among the three groups and upper and lower jaw P < 0.01, significant.

Sources of variation	Degree of freedom (df)	Sum of squares (SS)	Mean squares (MSS = SS/df)	F ratio = group MSS/error MSS
Between groups	2	199.58	99.79	624.65
Jaw	1	0.41	0.41	2.58
Error	188	30.03	0.15	-
Total	190	230.03	1.20	-

The health and diet of the rats appeared adequate, and all the animals gained a similar amount of weight during the experimental period. The difference in total caries rates among the groups was statistically significant (p < 0.01). There was no statistically significant difference in the caries rates between the maxilla and mandible in any group (p > 0.05). Rats consuming the cariogenic diet and non-fluoridated water had the highest rate of caries. Rats that consumed black tea had a significantly lower rate of caries than the non-fluoridated water group. Although tea consumption decreased the development of caries, the caries score in the group receiving tea was significantly higher than in the group receiving fluoridated water.

## Discussion

Previous studies had correlated the anticariogenic potential of tea with its fluoride content [14,15]; however, recent research has highlighted the role of several polyphenolic compounds, such as catechins, theaflavins, tannins, and flavonoids, present in tea for their anticariogenic activity [16]. The present study has been carried out on similar lines to a study by Touyz et al. [11], as both studies are of short duration and are carried out in small groups of experimental animals. However, the experimental animals used in both studies are different. Touyz et al.'s study [11] used Sprague-Dawley rats and the present study has been carried out on the more commonly available albino rats. The tea leaf used in Touyz et al.'s study [11] is of Tetley brand (which is an international brand) whereas, in the present study, we have used non-branded locally available Assam tea. Moreover, Touyz et al.'s study [11] is carried out in a more sophisticated and advanced laboratory facility in comparison to the present study.

The aim of this study was to ascertain whether black tea consumption affects the development of caries in young rats placed on a cariogenic diet. This experiment was carried out on 17-day-old albino rats. The animals were segregated into three categories of eight each and were placed on a cariogenic diet (MIT 200). Sucrose, fat, and albumin in the diet were constant in composition and low in ash content. This diet also contains a specially modified salt mixture that provides adequate and balanced amounts of all the minerals required by the rat and also permits the development of experimental caries. All the animals gained a similar amount of weight (about 6 g over two weeks). Touyz et al. also used a similar diet to induce caries in Sprague-Dawley rats. However, some researchers have suggested the use of a high-sucrose diet for inducing experimental caries [17,18].

A pure culture of *S. mutans* bacteria was used in this study. The bacterial inoculum was prepared by overnight incubation of a pure culture of *S. mutans*. This inoculum was further diluted and adjusted to McFarlane number 0.5 (i.e., 0.1 mL of the suspension contained 4 × 109 colony-forming units of *S. mutans*). This bacterial inoculum was administered at a concentration of 1 mL of inoculum in 100 mL of drinking water for five consecutive days [19,20].

It was observed that in Group I, most of the teeth showed moderate dentinal involvement, whereas, in Groups II and III, the teeth showed carious lesions of the enamel only. The mean value for the total caries score was 1.17, and that of Group III was 0.6. ANOVA was used to compare the total caries score between the three groups statistically. This revealed that there was a significant difference in the caries score between Groups II and III. It was also observed that the caries were limited to the enamel only in the group receiving fluoridated water and tea. The carious lesions involving the dentin were observed only in Group I. There were very few carious lesions on the proximal surfaces in Groups II and III. The findings of the present investigation were consistent with those reported by Touyz et al., who conducted the experiment on 18-day-old Sprague-Dawley rats.

In addition to its bactericidal effect against *S. mutans* and *Streptococcus sobrinus* [21], tea also inhibits glucosyltransferase, thus limiting the synthesis of sticky glucan [22]. The theaflavins in black tea have potent inhibitory activity against glucosyltransferase [23,24]. Numerous studies have suggested that drinking tea regularly may lessen the frequency and severity of dental caries in humans [25,26]. Studies suggest that oolong tea extract inhibits the adherence of *S. mutants* to the tooth surface [27,28]. The administration of oolong tea extract and its polyphenolic compound into diet 200 and drinking water resulted in a significant reduction in caries development and plaque accumulation in rats infected with mutans streptococci [29]. It has been reported that green tea catechins and black tea theaflavins can inhibit salivary amylase [30]. In fact, black tea theaflavins were reported to be more potent inhibitors of alpha-amylase [31]. It has been observed that when a solution of green tea extract containing eight catechins was used as a mouth rinse, each catechin was retained in mg/ml levels in saliva for up to 60 minutes [32]. These studies show that tea and its components interfere specifically with each of the steps of the pathogenesis of dental caries. In addition to these possible anticariogenic effects, tea also exerts a potential bactericidal effect on *S. mutans* and *S. sobrinus* [33,34]. The theaflavins present in black tea exert their antibacterial activity by damaging the bacterial cell membranes [35]. From this study, we can conclude that the intake of black tea can reduce the development of caries in young rats fed on a cariogenic diet. Similar results have been reported in other studies carried out on experimental animals [36,37]. The precise mechanism by which tea exerts its anticariogenic effect is not clear. It is speculated that because catechins have a known affinity for proteins, the interaction between catechins and related compounds with these proteins induces structural and functional changes that inhibit the adhesion of *S. mutans* [38].

The limitations of the study include a small sample size and a short duration of time. A bigger sample size and a longer period are required to arrive at a definite conclusion regarding the role of tea as an anticariogenic agent and the mechanism whereby tea influences cariogenesis.

## Conclusions

Ancient Japanese believed that drinking tea kept the mouth clean. Modern scientific studies have proven that tea has anticariogenic properties. Tea could prove to be a very cost-effective public health intervention because eliminating the causal agent is a crucial step in the prevention of dental caries. From the above experimental observations, it can be concluded that consumption of black tea reduced the development of dental caries in young rats fed on a cariogenic diet. Because the water used for preparing tea was fluoride-free, we can assume that the anticavity activity of black tea is because of certain constituents such as catechins, theaflavins, tannins, and flavonoids of tea other than fluoride.
